# Determination of Ochratoxin A (OTA), Ochratoxin B (OTB), T-2, and HT-2 Toxins in Wheat Grains, Wheat Flour, and Bread in Lebanon by LC-MS/MS

**DOI:** 10.3390/toxins11080471

**Published:** 2019-08-12

**Authors:** Jomana Elaridi, Osama Yamani, Amira Al Matari, Saada Dakroub, Zouhair Attieh

**Affiliations:** 1 Department of Natural Sciences, School of Arts and Sciences, Lebanese American University, P.O. Box 13-5053, Beirut 1102-2801, Lebanon; 2 Department of Laboratory Science and Technology, American University of Science and Technology, Achrafieh 16-6452, Lebanon

**Keywords:** Ochratoxin A, ochratoxin B, T-2 toxin, HT-2 toxin, wheat, flour, bread, Lebanon

## Abstract

Cereals are prone to fungal infection during growth, harvesting, transportation, and/or storage. As a result, cereals such as wheat grains and wheat-derived products may be contaminated with mycotoxins leading to acute and chronic health exposure. The current study investigated the presence of the mycotoxins: ochratoxin A (OTA), ochratoxin B (OTB), T-2, and HT-2 toxins in samples of wheat grains (*n* = 50), wheat flour (*n* = 50), and bread (*n* = 37) from the main mills in Lebanon using LC-MS/MS. Accuracy ranged from 98–100%, recoveries from 93–105%, and intraday and interday precision were 5–7% and 9–12%, respectively. The tested wheat grains, wheat flour, and bread samples did not contain detectable levels of T-2 and HT-2 toxins and OTB. Four wheat flour samples (8% of flour samples) showed positive OTA levels ranging from 0.6–3.4 μg·kg^−1^ with an arithmetic mean of 1.9 ± 0.2 μg·kg^−1^. Only one sample contained an OTA concentration greater than the limit set by the European Union (3 μg·kg^−1^) for wheat-derived products. This study suggests that mycotoxin contamination of wheat grains, wheat flour, and bread in Lebanon is currently not a serious public health concern. However, surveillance strategies and monitoring programs must be routinely implemented to ensure minimal mycotoxin contamination of wheat-based products.

## 1. Introduction

Mycotoxins are amongst the most prominent and dangerous toxins associated with food safety. Mycotoxins are poisonous secondary metabolites produced by different filamentous fungi such as those of the genus *Aspergillus*, *Penicillium*, and *Fusarium*. Mycotoxins can have harmful acute and chronic effects and ultimately threaten human and animal health. Reportedly, 25% of the world’s food crops are contaminated with mycotoxins, indicating that it is a persistent worldwide problem [1,2,3,4,5,6]. Ochratoxins, a specific class of mycotoxins, cause renal nephropathy, acute renal failure, lesions, and acute tubular necrosis in humans [7,8]. Ochratoxin A (OTA) is one of the most known and prevalent mycotoxins and is classified as a possible human carcinogen (Group 2B) by the International Agency for Research on Cancer (IARC) [9]. *Aspergillus ochraceus*, a mold species known to produce OTA and ochratoxin B (OTB), the dechlorinated version of OTA, is a contaminant in many staple food commodities including wheat grains, wheat berries, and cereals, and typically colonizes in temperate and tropical geographical areas [10,11].

Trichothecene mycotoxins are naturally occurring byproducts produced by more than 350 species of fungi and are pathogenic to animals and humans. Fusarium head blight (FHB) is a destructive disease affecting cereal grain crops that is caused by a series of trichothecene-producing *Fusarium* species [12,13]. FHB outbreaks have been reported in England, America, Asia, Europe, and South Africa, and have led to substantial economic losses for farmers worldwide [14,15]. T-2 mycotoxin is one of the most toxic and most extensively studied trichothecenes [16]. T-2 toxin and its main metabolite, HT-2 toxin, are non-volatile and highly resistant to heat and UV light [16,17]. T-2 and HT-2 are toxic to all animal species. Some adverse effects include a decrease in blood cell and leukocyte count, reduction in plasma glucose, and pathological changes in the liver and stomach [18]. T-2 toxin is also associated with alimentary toxic aleukia, deoxyribonucleic acid damage, and induction of apoptosis [18,19].

The most common route of human exposure to mycotoxins is *via* dietary intake of important agricultural products such as wheat, maize, and oats [1,20,21,22]. Wheat is grown on the greatest land area of all commercial crops and is one of the most important food grains and cereal sources worldwide. Wheat is a major component in the diets of people around the world due to the ease of grain storage and the ability to convert it into flour then into bread. Fungal contamination of wheat crops typically occurs during growth, harvesting, or post-harvesting while in storage or during processing especially under environmental conditions of heat and/or humidity (moisture) [23,24]. Mycotoxins may be present in food products even when the mold is not visible and despite the implementation of good agricultural, storage, and processing practices [1]. Therefore, surveillance and sampling for detection, and quantification are essential for the management of mycotoxins in all commodities. Routine laboratory analysis for ochratoxins and tricothecenes in various food products is performed by Enzyme-Linked Immuno Sorbent Assay (ELISA), high-performance liquid chromatography (HPLC) coupled with a fluorescence (FLD), ultraviolet (UV), diode array (DA), or mass spectrometry (MS) detector or gas chromatography (GC) combined with an MS, flame ionization (FI), or electron capture (EC) detector [25,26,27,28,29]. There is a huge abundance of literature related to the presence of mycotoxins in food and feedstuff. In the last decade, studies from various countries including Canada [30], Serbia [3,31,32,33,34], Portugal [11,35], Russia [36], Romania [37,38], Belgium [39], Italy [40,41], Spain [42,43,44], Germany [45], Poland [46], Sweden [47], Croatia [48], Lithuania [49], South Korea [50], Japan [51], Turkey [52,53], Brazil [54,55], the UK [56,57], and several African countries [58,59] have reported the presence of ochratoxins and tricothecenes in wheat, flour and/or bread. In Lebanon, literature on mycotoxin contamination of cereals is scarce [60,61,62,63]. In 2016, however, the Ministry of Public Health (MoPH) released a statement declaring that samples of wheat grains imported to Lebanon contained dangerously high levels of OTA [64,65,66] – an announcement that incited immense public concern. Apparently, five of twelve samples of a wheat batch from Russia showed OTA levels of 26 μg·kg^−1^, violating the maximum level of 5 μg·kg^−1^ [64,65,66]. The Ministry of Economy and Trade denied these claims stating that wheat grains recently imported into the country were carcinogen-free. Several months later, the MoPH indicated that wheat imports to Lebanon were safe [67] but only after alarming the population with scandalous claims of wheat contamination.

A plethora of studies have investigated the occurrence of ochratoxin A, T-2, HT-2, and/or other mycotoxins in samples of wheat (grains and berries), wheat flour, and bread all over the world. Most recently, a study in Algeria revealed that OTA contaminated 69.2% of wheat grains and 92.8% of wheat-derived products. OTA concentrations ranged from 0.21–27.31 μg·kg^−1^ and 0.84–34.75 μg·kg^−1^, respectively, with 50% of positive samples exceeding the EU maximum limit [58]. This contamination was attributed to high temperatures and humidity storage conditions which favor the formation of *Aspergillus* fungi in addition to poor manufacturing practices during processing. An extensive study conducted from 2004–2016 in Serbia on cereal and dairy products reported that T-2/HT-2 and OTA were detected in 45.5%, and 28.1% of samples respectively [3]. Another study which analyzed different types of flour including wheat, buckwheat, rye, oat, barley, rice, and millet flours, marketed in Serbia showed that ochratoxin A (OTA) was found in 29% of flour samples [31]. In 2018, a report emanating from Kosovo revealed that 9% of wheat flour samples were contaminated with OTA with concentrations ranging from 0.26–0.85 μg·kg^−1^ [68]. A five-year-long study conducted between 2009–2014 in Canada revealed that OTA was detected in more than 50% of wheat-based products specifically wheat, oats, milled products of other grains [30]. Concentrations ranged from 0.040 to 631 μg·kg^−1^. In 2016, fifty-five processed grain products were analyzed in Japan. HT-2 was detected in six samples, and T-2 was detected in two samples with concentrations of less than 20 μg·kg^−1^ [69].

Importantly, no study has been conducted in Lebanon to reveal the concentrations of ochratoxins and tricothecenes in wheat grains, wheat flour, and bread using advanced analytical techniques such as tandem liquid chromatography-mass spectrometry (LC-MS/MS). Thus, the aim of the present study was to use LC-MS/MS for evaluating the occurrence and accurately measuring the concentrations of OTA, OTB, T-2, and HT-2 toxins in different wheat grains, wheat flour, and bread samples available in Lebanon’s main mills and bakeries. 

## 2. Results and Discussion

A total of 137 samples of wheat grains (n = 50), wheat flour (n = 50), and bread (n = 37) were analyzed for ochratoxin A (OTA), ochratoxin B (OTB), T-2 toxin, and its metabolite HT-2 toxin using LC-MS/MS. The various wheat grains and wheat flour samples were collected from the nine major mills which are responsible for the distribution of imported wheat and flour to bakeries, supermarkets, and patisseries all over the country. The bread samples were randomly obtained from bakeries and supermarkets in the greater Beirut area. 

The samples were subjected to standard pre-treatment liquid-liquid extraction methods [29]. Multiple extraction solvents were initially attempted, including various mixtures of MeCN and MeOH, MeCN and water, MeOH and water, and MeCN and acetic acid. The solvent mixture ultimately chosen was 95% MeCN: 5% formic acid because it resulted in the clearest supernatant after centrifugation and optimal extraction yields (>90%) for each of the analytes. The presence of formic acid may have improved extraction yields by breaking interactions between the mycotoxins and other sample constituents, such as proteins and carbohydrates [26].

The validity of the method was evaluated based on the accuracy, intra- and inter-day precision, linearity, and sensitivity (limit of detection (LOD) and limit of quantification (LOQ)). The linearity of the method was assessed by analyzing OTA, OTB, T-2, and HT-2 in selected concentration ranges with multiple calibration points. The data for peak area were treated by linear regression analysis and linearity was expressed as a coefficient of determination (R^2^). All correlation coefficients were over 0.998. The LOD/LOQ values were 0.12/0.35 ng·mL^−1^ for OTA, 0.06/0.18 ng·mL^−1^ for OTB, 0.39/1.19 ng·mL^−1^ for T-2 toxin, and 0.83/2.52 ng·mL^−1^ for HT-2 toxin. A certified blank wheat sample (BCR47) was analyzed in addition to spiked reagent blank, sample duplicates, and spiked samples as part of analytical quality control. For precision and accuracy, spiked samples (n = 3) were analyzed three times a day (intraday) and for five days (interday) to ensure reproducibility. Accuracy was between 98–100% and intraday and interday precision were 5–7% and 9–12%, respectively. Sample spike recoveries were assessed by spiking the certified blank sample with different concentrations and extracted using the same method and calculated using the same principle of routine quantitation. The use of LC-MS/MS enabled the simultaneous identification and quantification of the mycotoxins: OTA, OTB, T-2, and HT-2 toxin and recoveries ranged from 93–105% for OTA, OTB, T-2, and HT-2 toxins (Table 1). Calibration curves, chromatograms and data related to the method are found in the Supplementary Materials.

Our study revealed a very low occurrence of the mycotoxins: OTA, OTB, T-2, and HT-2 in wheat grains and the wheat-derived products of flour and bread. Importantly, the tested wheat grains, wheat flour, and bread samples did not contain detectable levels of T-2 and HT-2 toxins and OTB. Four wheat flour samples (8% of flour samples) showed positive OTA levels ranging from 0.6 μg·kg^−1^ to 3.4 μg·kg^−1^ (Table 2), with an arithmetic mean of 1.9 ± 0.2 μg·kg^−1^. One of the samples contained an OTA level greater than the limit of 3 μg·kg^−1^ set by the European Union for wheat-derived products [70]. All samples did not exceed the OTA contamination threshold (5 μg·kg^−1^) set by the Lebanese MoPH. The four contaminated wheat flour samples came from three different mills around the coastal Beirut area. We attribute the presence of OTA in these flour samples to the hot and humid storage and processing conditions in the mills.

Our results contradicted news released by the MoPH in 2016 claiming the presence of carcinogenic mycotoxins in wheat grains and bread [64,65,66]. We suspect that the discrepancy between the results reported by the Ministry and those observed in this study may be due to the use of different and less sensitive analytical procedures and equipment compared to the LC-MS/MS method used here. It is difficult to ascertain the integrity of the Ministry’s statement as results were not published in a peer-reviewed journal and the analytical method was not disclosed. It is most likely that an advanced analytical technique was not utilized due to the limited resources and facilities available in Lebanon. LC-MS/MS is undoubtedly one of the most sensitive, selective, and accurate analytical techniques for the measurement of analytes in various matrices. A stringent quality assurance and quality control was performed in this study to confirm reliability. We acknowledge that we cannot directly compare our results to those of the Ministry as we analyzed samples collected at different times than the ones reported by the Ministry and used a different analytical technique. Nonetheless, no other study has since investigated the occurrence of mycotoxins in these staple food products.

In Lebanon, only scarce studies relating to food contamination and dietary exposure to mycotoxins are available [60,61,62,63]. In fact, only two reports in the literature investigated the detection of mycotoxins in wheat in Lebanon [60,62]. In 2011, Joubrane et al. performed a thorough survey of filamentous fungi extracted from 156 samples of wheat cultivated across the Bekaa region of Lebanon [62]. OTA was detected in 23.7% of local wheat samples in concentrations above 3.0 μg·kg^−1^ and this was attributed to cultivation practices, the high temperatures and humid climate and storage conditions [62]. In a 2004 study, blood plasma samples drawn from healthy individuals and wheat samples were analyzed for OTA. OTA was detected in 33% of the tested plasma samples with concentrations ranging from 0.10 to 0.87 ng·mL^−1^ revealing significant exposure of Lebanese consumers to OTA [60]. OTA detection was observed in 12% of wheat samples with a mean concentration of 0.15 μg·kg^−1^ [60]. In these studies, HPLC-FLD, a less sensitive technique was employed for the analysis of locally grown wheat [62] and wheat obtained from markets across the country and Beirut silos [60]. Importantly, local wheat production accounts for only 10% of the wheat consumed in Lebanon [71], while 90% of the consumed wheat across the country is imported. In this study, the use of LC-MS/MS and the analysis of imported wheat grains and wheat flour samples from the major mills in Lebanon offers a more reliable and comprehensive investigation. In addition, T-2 and HT-2 toxins were not investigated in any of the aforementioned studies.

The Lebanese government has since ensured that high quality and non-contaminated wheat is being imported from countries such as Russia and Ukraine which are amongst the top wheat-exporting countries in the world. In fact, of the 625,661 tons of wheat that were imported into Lebanon in 2015, 530,070 tons (85%) came from Russia, Ukraine, Moldova, and Latvia [71]. The origin of the analyzed wheat grain samples in this study is not definitively known, however, it is presumed that they emanated from the aforementioned countries. Although we did not find studies investigating mycotoxin contamination of wheat from Ukraine, Moldova, and Latvia, several studies exploring the presence of mycotoxins in Russian crops revealed detectable levels of mycotoxins in wheat samples. Specifically, in 2013, Tutelyan et al. reported food grain contamination with fusariotoxins: deoxynivalenol (DON), zearalenone (ZEN), fumonisins (FB1 and FB2), and T-2 and HT-2 toxins [36]. Harvests of 2005-2010 in different regions of Russia were investigated by performing ELISA and LC-MS/MS analysis which revealed the presence of T-2 toxin and HT-2 toxin in 14% and 17% of samples, respectively, though maximum levels of the two toxins were not exceeded [36]. The higher occurrence of analyzed mycotoxins in wheat in Russia compared to the wheat tested in this study is expected due to the fact that wheat imported from Russia, or from any country for that matter, is tested prior to shipment from the country of origin to protect the international trade of wheat exportation. 

Importantly, the OTA contamination of wheat flour samples—rather than wheat grains—in our investigation, also highlights the necessity for better surveillance of storage and processing environments of wheat flour samples. The effective drying of grains, cereals, and food products has proven to be the most feasible and effective method for minimizing mycotoxin production [72]. Thus, we presume the wheat flour samples were contaminated primarily due to the humid and high temperatures associated with the storage and/or processing they have been subjected to.

In the present study, bread samples showed no detectable concentrations of OTA, OTB, T-2, and HT-2 toxins. In general, mycotoxins are thermally stable and survive conditions associated with the processing of contaminated foods including cooking, baking, roasting, and sterilization [1,24,73]. Karlovsky et al. reported that food processing can reduce mycotoxin exposure by eliminating mycotoxins, transforming them into less toxic derivatives or by reducing their bioavailability but complete elimination of mycotoxins from food products by processing is rarely completely efficient [73].

## 3. Conclusions

This study is the first in Lebanon to utilize the sensitive analytical technique of LC-MS/MS for quantitation of mycotoxins: OTA, OTB, T-2, and HT-2 toxin in wheat grains, wheat flour, and bread. In the current study, wheat, flour, and bread samples were determined to be free of OTB, T-2, and HT-2 toxin though 8% of flour samples were found to be contaminated with ochratoxin A. Only 1 wheat flour sample (2%) was contaminated with an OTA level greater than the limit set by the European Commission (3 μg·kg^−1^). OTA, OTB, T-2, and HT-2 were not detected in wheat grains and bread. Fortunately, our investigation revealed little or no contamination of these toxins which may quench public concern regarding the integrity of wheat and wheat-derived products. The sources of imported wheat grains are evidently dependable and the applied storage conditions are satisfactory. However, the presence of OTA in wheat flour indicates that there are conditions, such as heat and humidity, that promote fungal growth in the harvesting, processing, or storage stages of food production. Thus, continuous nationwide monitoring must be implemented to all Lebanese mills and surveillance should be expanded to a broader range of mycotoxins to ensure the quality of wheat, flour, and bread which are staples in the Mediterranean diet.

## 4. Materials and Methods 

### 4.1. Reagents and Chemicals

All reagents and chemicals used were of analytical HPLC-grade. Methanol (MeOH), formic acid, and acetonitrile (MeCN) were obtained from Fisher-Scientific and used in the extraction and preparation of the mobile phase. High purity deionized water was generated using Direct-Q^®^ UV Millipore, T-2 toxin-^13^C_24_ solution (25 μg·mL^−1^ in MeCN) and ochratoxin A-^13^C_20_ solution (10 μg·mL^−1^ in MeCN), T-2 toxin solution (100 μg·mL^−1^ in MeCN), HT-2 toxin solution (100 μg·mL^−1^ in MeCN), ochratoxin A (10 μg·mL^−1^ in MeCN), ochratoxin B (10 μg·mL^−1^ in MeCN), and certified blank wheat sample (BCR471) were obtained from Sigma-Aldrich.

### 4.2. Sample Collection and Preparation

Representative samples of wheat grains, wheat flour, and bread were collected in collaboration with The Consumer Protection Directorate of the Ministry of Economy and Trade. Fifty samples of wheat grains and fifty samples of wheat flour were randomly collected from the nine main mills distributed in different areas in Lebanon. The wheat samples were drawn from the stock that was ready for milling. Approximately 500 g of each sample was placed in a tightly sealed sterile plastic bag. In addition, a total of thirty-seven Arabic bread samples were collected from various bakeries distributed across the greater Beirut area. All samples (137 total) were directly transported to the laboratory and stored in a dry room.

### 4.3. Preparation of Internal and External Standards

A calibration curve was prepared for each batch of samples. This required the preparation of several standard solutions of different concentrations. Standards were used to prepare three main solutions: (*i*) Multi-stock solution, (*ii*) multi-working solution, and (*iii*) internal standard solution. The multi-stock solution was prepared by mixing specific volumes as shown in Table 3 of each external standard solution and diluting to 100 mL with MeOH. The multi-working solution was made by diluting 310 µL of the multi-stock solution with MeOH to a final volume of 5.0 mL. Characteristics of the multi-stock and multi-working solution are presented in Table 3.

For the internal standard solution, 100 µL of OTA-^13^C_20_ and 400 μL of T-2 toxin ^13^C_24_ solution were placed in a vial and diluted with MeOH to a volume of 20 mL. The final concentration of the internal standard solution was 0.5 μg·mL^−1^ in MeCN. For each tested sample, 25 µL of the solution was used.

### 4.4. Extraction of Mycotoxins

An MeCN:formic acid solution (20 mL, 95%:5%) was added to each falcon tube containing 5.00 g of the sample (wheat and bread samples were finely grounded). The mixtures were shaken well then sonicated for one hour with shaking every 5 minutes. The falcon tubes were centrifuged for 4 minutes at 3000 rpm.

### 4.5. Calibration and Sample Preparation for Analysis and Quality Control

For calibration and sample analysis, 1 mL of the supernatant was placed in an evaporation tube, and in another 6 tubes used for calibration, an aliquot (50, 350, 650, 950, 1250, 1550 μL) was pipetted into each tube. Then 25 μL of the internal standard solution (0.5 μg·mL^−1^) was added to each sample, vortexed and evaporated to dryness using nitrogen gas. The samples were reconstituted in two stages: the first stage, by adding 250 μL of MeOH with 10% formic acid (mobile phase B), mixing using a vortex and sonication for 2 minutes prior to the addition of 250 μL deionized water with 10% formic acid (mobile phase A), then the sample was transferred to a vial ready for injection. 

### 4.6. Method Validation

Analytical quality control was assessed by analysis of certified blank wheat sample (BCR471) and spiked reagent blank to ensure no contamination from the reagents and the vessels in addition to sample duplicates and spikes. Reagent blanks contained undetectable concentrations of the mycotoxin analytes. The method was validated by determining the linearities, average recoveries, sensitivities (LOD, LOQ, method detection limit (MDL), and method quantitation limit (MQL)). The LOD was calculated as 3.3 times the standard deviation of intercept divided by the slope of the calibration curve constructed using 4 low concentrations as recommended by the International Council for Harmonization of Technical Requirements for Pharmaceuticals for Human Use (ICH) guidelines [74]. The LOQ was calculated in a similar manner but it is 3 times the LOD (Table 3), which reflects the instrument’s sensitivity. The LOQ and LOD values were converted to MDL and MQL (in ng·g^−1^ of sample) by multiplication of the value with the D.F. = 4 (Table 3). Accuracy (%), precision (RSD%), and matrix effects were also assessed. Accuracy (bias) and precision were measured by spiking 2 levels: one equal to the second point of the calibration curve and the other equal to point number 5 of the calibration curve. This experiment was repeated for 5 days. Then ANOVA analysis was conducted to calculate the MS (Multiple Square error) for between and within the group. The precision for interday and intraday reproducibility was calculated where the highest value is reported either if it’s for low level or high level. Bias was calculated based on the percentage deviation of grand mean of 5 days reading to the nominal concentration. Then accuracy was calculated by addition of the bias results to value of 100.

For matrix effect, the certified blank sample (wheat) was extracted and after extraction, the 1.0 mL that was routinely used, was evaporated. The standard (STD) and internal standard (ISTD) were added after drying. The same amounts of STD and ISTD were added to another clean tube (neat). This experiment was repeated 6 times, then the percentage of each mycotoxin STD and ISTD response in matrix tube to neat tube was calculated, and also the area R in both tubes in addition to the % of area ratio in matrix tube to area ratio in the neat tube. Negative results were an expression of suppression and positive results were an expression of enhancement results. Results show ignorable effect in the area R part, which is the routine case, and for absolute area of the analyte or ISTD, the effect was 10–15% suppression.

Linearity (calibration models) were assessed based on a linearity test for each mycotoxin. The model selection was based on testing the residual error, and standardized residual error distribution. Linear model with trial of 4 weighting (1/x, 1/x^2^, 1/y, 1/y^2^) and Quadratic Model with trial of 4 weighting (1/x, 1/x^2^, 1/y, 1/y^2^) were assessed for residual error and standardized residual error distribution among different level concentrations and the model with least error with random scatter of standardized residual error was selected for each compound. The calibration curve used in the linearity test was the same point of routine calibration level but each point was replicated 6 times. Final models used were: OTA: linear no weighting; OTB: linear no weighting; T-2: quadratic, no weighting; HT-2: Quadratic x^2^, weighting. The coefficients of determination (R^2^) were all found to be >0.998.

### 4.7. LC-MS/MS Analysis

LC-MS/MS analysis was carried out using a Triple Quadrupole LC-MS/MS Mass Spectrometer (Sciex) equipped with an AB Sciex API 4000 system (AB Sciex, Framingham, MA, USA). Fronted with Shimadzu high performance liquid chromatography (HPLC) composed from solvent delivery pump series LC20AD with built-in low-pressure gradient mixer, auto-sampler series SIL20A, and column oven series CTO20AC. The autosampler temperature and injection volume were 25 °C and 10 μL respectively. Mobile phase A: 400 mL deionized water + 800 μL ammonium formate (5.0 M) + 400 μL formic acid (0.1%); Mobile phase B: 400 mL MeOH + 400 μL formic acid (0.1%). Equilibrium time: 2.00 min; Injection Volume: 10.00 µL; Pumping Mode: Low pressure gradient. Flow rate: 0.3000 mL·min^−1^; Column temperature: 40 °C; pressure range: 0–200 bar. Analytical column: Atlantis^®^ T3 C18, L = 15 cm, ID = 0.2, particle size 5.0 μm. 

## Figures and Tables

**Table 1:** Sensitivity data, accuracy, precision and recovery data for ochratoxin A (OTA), ochratoxin B (OTB), T-2, and HT-2.

Test	OTA	OTB	T-2	HT-2
LOD (ng·mL^−1^)	0.12	0.06	0.39	0.83
LOQ (ng·mL^−1^)	0.35	0.18	1.19	2.52
MDL (μg·kg^−1^)	0.48	0.24	1.56	3.32
MQL (μg·kg^−1^)	1.40	0.72	4.76	10.1
Accuracy (%)	100	98.8	100	100
Intraday RSD (%)	6.1	7.2	4.9	5.4
Interday RSD (%)	11.5	12.2	10.4	9.1
Recovery (%)	97	93	105	100

RSD = relative standard deviation.

**Table 2:** Ochratoxin A (OTA), ochratoxin B (OTB), T-2 and HT-2 in wheat, flour and bread samples.

Mycotoxin	Food	*n*	*n*(%)	Mean ± SD (μg·kg^−1^)	Minimum (μg·kg^−1^)	Median (μg·kg^−1^)	Maximum (μg·kg^−1^)
OTA	Wheat	50	-	-	-	-	-
	Flour	50	4 (8)	1.9 ± 0.2	0.6	2.1	3.4
	Bread	37	-	-	-	-	-
OTB	Wheat	50	-	-	-	-	-
	Flour	50	-	-	-	-	-
	Bread	37	-	-	-	-	-
T-2	Wheat	50	-	-	-	-	-
	Flour	50	-	-	-	-	-
	Bread	37	-	-	-	-	-
HT-2	Wheat	50	-	-	-	-	-
	Flour	50	-	-	-	-	-
	Bread	37	-	-	-	-	-

*n*: number of samples; n (%): number (percent) of positive samples (>LOQ), SD: standard deviation.

**Table 3:** Preparation of multi-stock and multi-working solutions.

Multi-stock Solution				Multi-working Solution
Toxin	V_1_ *(μL)	C_1_ *( μg·mL^−1^)	C_2_^†^ (μg·mL^−1^)	C_3_^‡^ (ng·mL^−1^)
T-2 toxin	0.3	100	0.3	19
HT-2 toxin	0.5	100	0.5	31
OTA	0.3	10	0.05	3.1
OTB	0.3	10	0.05	1.9

* C_1_,V_1_ = initial concentration and volume of external standard, **^†^** C_2_ = new concentration of each external standard after dilution with MeOH (V_T_ = 100 mL). **^‡^** C_3_ = 310 μL of multi-stock solution diluted to 5.0 mL with MeOH.

## Supplementary Materials

The following are available online at https://www.mdpi.com/2072-6651/11/8/471/s1, LC-MS/MS method optimization, Figure S1: Chromatograms of mycotoxin standards; Figure S2: OTA calibration curve for method linearity; Figure S3: OTB calibration curve for method linearity; Figure S4: T-2 toxin calibration curve for method linearity; Figure S5: HT-2 toxin calibration curve for method linearity; Figures S6 and S7: Chromatograms of positive samples.
