# Supplementary material for: Determination of Ochratoxin A (OTA), Ochratoxin B (OTB), T-2, and HT-2 Toxins in Wheat Grains, Wheat Flour, and Bread in Lebanon by LC-MS/MS
Source: Toxins (Basel). 2019 Aug 12;11(8):471. doi: 10.3390/toxins11080471 (PMC6723938; doi:10.3390/toxins11080471)
Supplement: Supplementary file 1 [file toxins-11-00471-s001.pdf]

Supplementary Information for: Determination of Ochratoxin A (OTA), ochratoxin B (OTB), T-2 and HT-2 toxins in wheat, flour and bread in Lebanon by LC-MS/MS

Jomana Elaridi, Osama Yamani, Amira Al Matari, Saada Dakroub and Zouhair Attieh

LC-MS/MS method optimization: Method optimization was initiated with full-scan experiments using the ESI interface in both positive and negative modes by infusion of solutions of OTA, OTB, T-2 toxin and HT-2 toxins *via* syringe pump to select the most suitable precursor ions. All toxins generated higher precursor ion signal intensities or better fragmentation patterns (i.e., more suitable characteristic productions for multiple reaction monitoring (MRM) detection) in positive ion mode. These findings were in agreement with other analytical methods which also detected T-2 and HT-2 toxin *via* LC-MS/MS [1–3]. Final gradient program is described as mobile phase A : mobile phase B, run time: 60:40, 0.01-3.00 min; 5:95, 3.00-7.10 min; 60:40, 7.10-10.00 min. MRM parameters used for analysis are shown in Table 1.

Table 1. LC-MS/MS MRM Parameters

Mycotoxin	Q1	Q3	DP (V)	CE (V)
OTA	404	239/358	40	30/19
OTB	370	159/324	40	55/20
T2	484	215/305	40	25/18
HT-2	442	263/215	40	18/18

DP: Declustering potential , CE: Collision energy

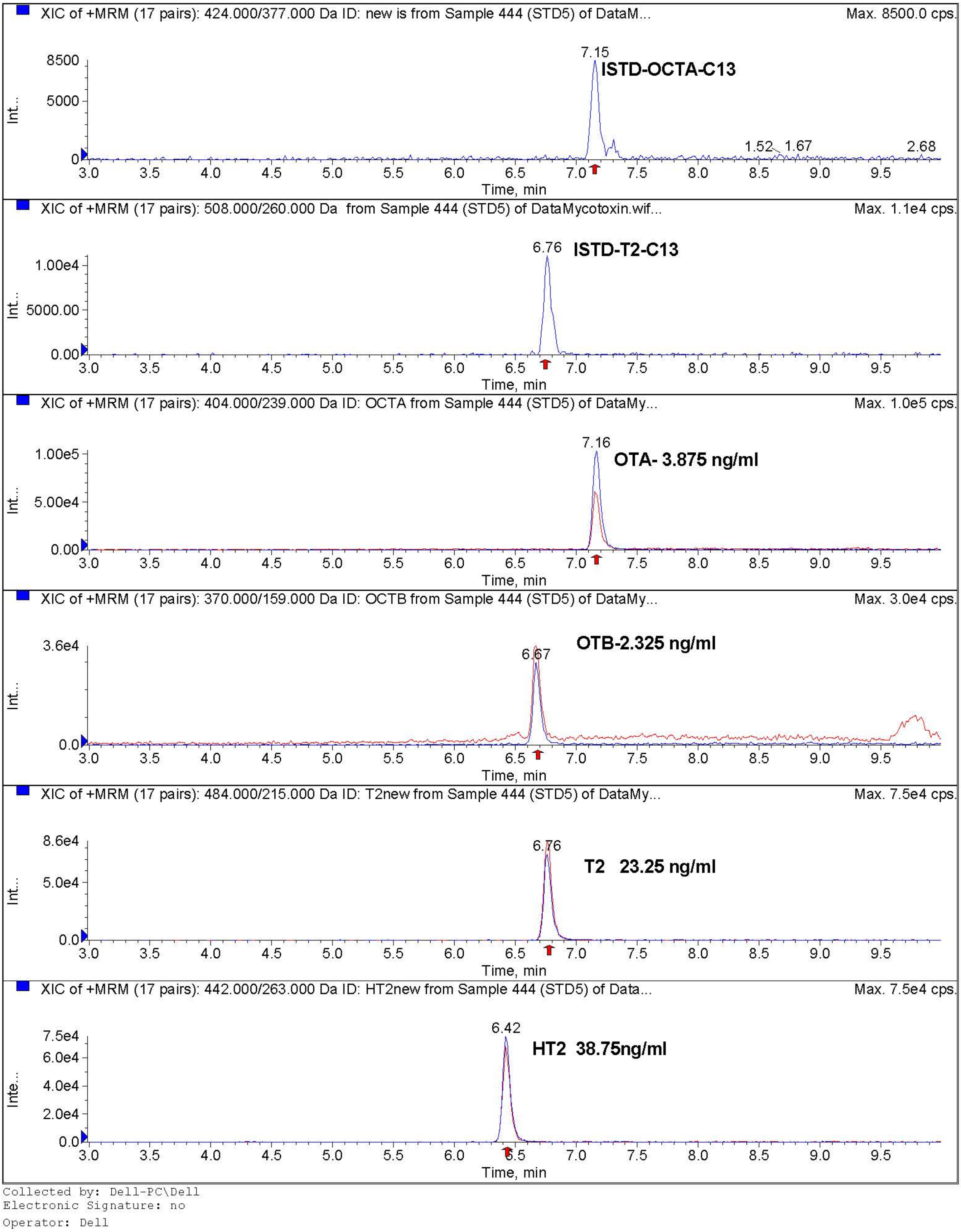

Figure S1. Chromatograms of mycotoxin standards

Analyte Name: OCTA
Internal Standard: OCTAc13

Data File	DataMycotoxin.wiff	Result Table	linearity-myco.rdb
Acquisition Date	6/8/2017 12:35:18 AM	Algorithm Used	IntelliQuan
Acquisition Method	mycodraft2.dam	Instrument Name	API 4000
Project	Default		

Regression Equation: y = 3.94 x + 0.00518 (r = 0.9985)

Expected Concentration	Number of Values	Mean Calculated Concentratio	% Accuracy	Std. Deviation	%CV
0.155	6	0.16	106.0	0.01	6.3
1.09	6	1.08	99.1	0.06	5.7
2.01	6	1.99	99.0	0.09	4.6
2.94	6	2.94	100.0	0.11	3.8
3.88	6	3.93	101.3	0.13	3.3
4.8	6	4.77	99.3	0.09	2.0

Printing Time: 01:19:44 AM
Printing Date: Tuesday, July 30, 2019

linearity-myco.rdb (OCTA): "Linear" Regression ("No" weighting): y = 3.94 x + 0.00518 (r = 0.9985)

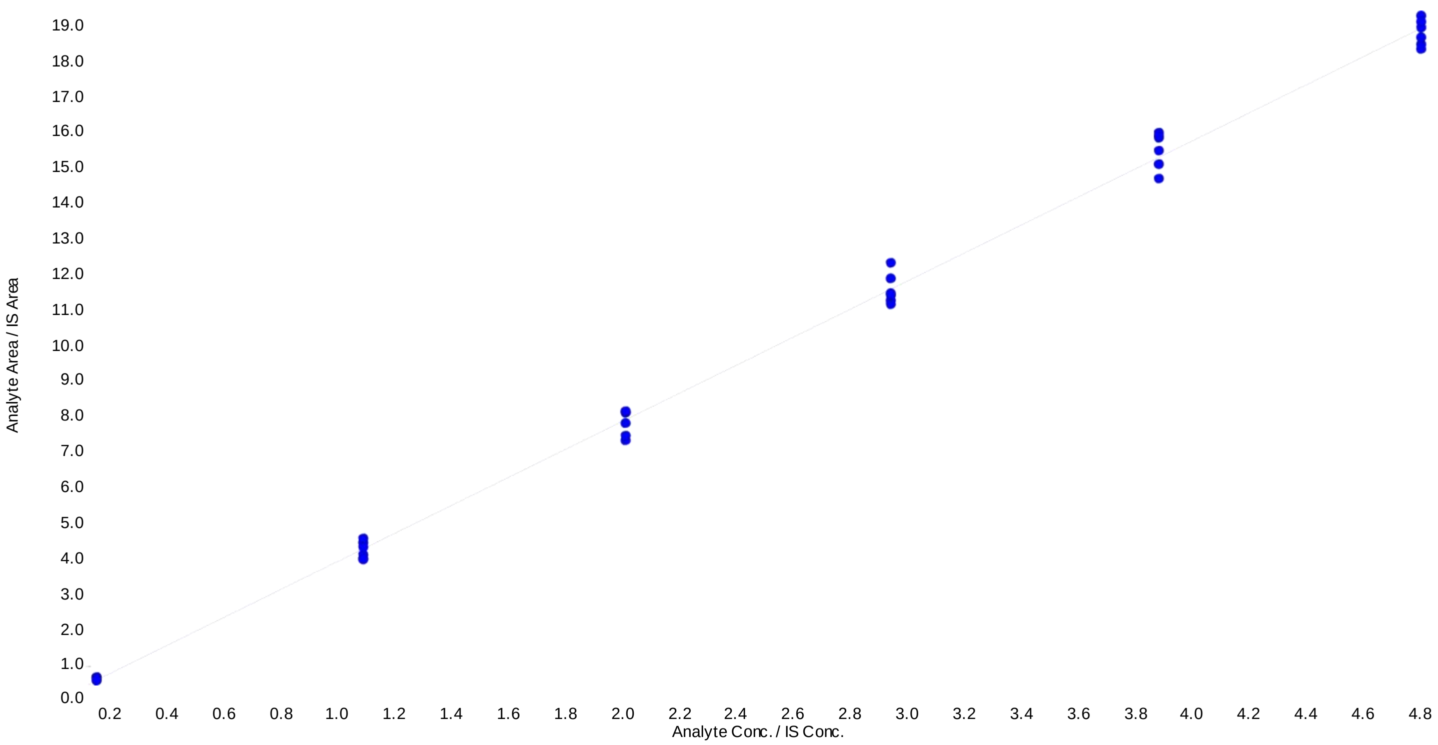

Collected by: N/A
Electronic Signature: no
Operator: Dell

Figure S2. OTA calibration curve for method linearity.

Analyte Name: OCTB
Internal Standard: OCTAc13

Data File	DataMycotoxin.wiff	Result Table	linearity-myco.rdb
Acquisition Date	6/8/2017 12:35:18 AM	Algorithm Used	IntelliQuan
Acquisition Method	mycodraft2.dam	Instrument Name	API 4000
Project	Default		

Regression Equation: y = 5.05 x + 0.1 (r = 0.9989)

Expected Concentration	Number of Values	Mean Calculated Concentratio	% Accuracy	Std. Deviation	%CV

0.093	6	0.09	93.1	0.01	6.4
0.651	6	0.64	98.9	0.03	4.9
1.21	5	1.22	100.8	0.04	3.2
1.77	6	1.76	99.7	0.02	1.2
2.33	6	2.37	101.5	0.06	2.4
2.88	6	2.85	99.0	0.07	2.4

Printing Time: 01:21:13 AM
Printing Date: Tuesday, July 30, 2019

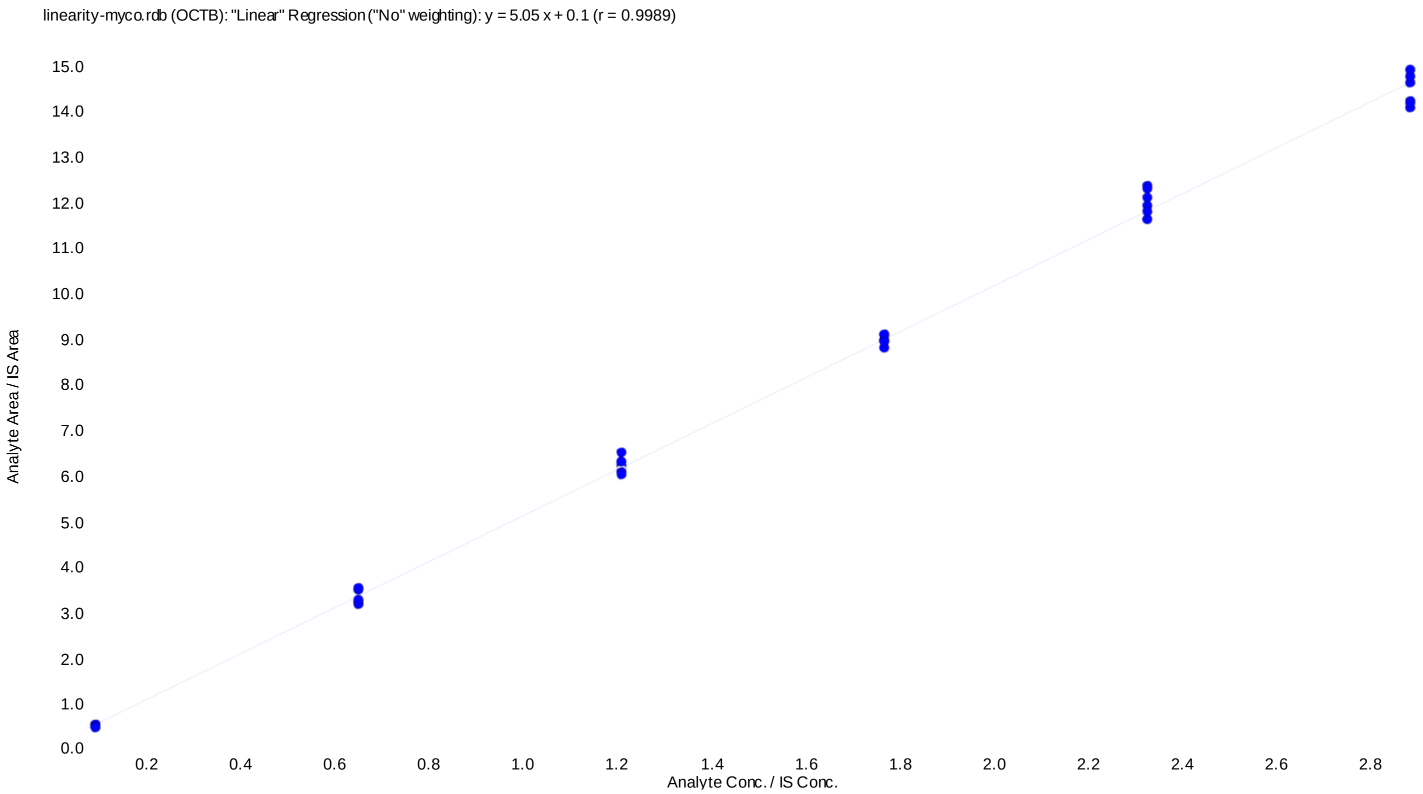

Figure S3. OTB calibration curve for method linearity.

Analyte Name: T2 NH4+

Internal Standard: T2-C13MH4+

**Data File**	DataMycotoxin.wiff	**Result Table**	linearity-myco.rdb
**Acquisition Date**	6/8/2017 12:35:18 AM	**Algorithm Used**	IntelliQuan
**Acquisition Method**	mycodraft2.dam	**Instrument Name**	API 4000
**Project**	Default		

Regression Equation: y = -0.000352 x^2 + 0.337 x + -0.00296 (r = 0.9993)

**Expected Concentratio n**	**Number of Values**	**Mean Calculated Concentratio**	**% Accuracy**	**Std. Deviation**	**%CV**
0.93	6	0.95	101.8	0.04	3.8
6.51	6	6.42	98.6	0.17	2.7
12.1	6	12.20	100.9	0.40	3.3
17.7	6	17.73	100.2	0.34	1.9
23.3	6	23.08	99.1	0.54	2.3
28.8	6	28.90	100.4	0.46	1.6

Printing Time: 01:23:42 AM
Printing Date: Tuesday, July 30, 2019

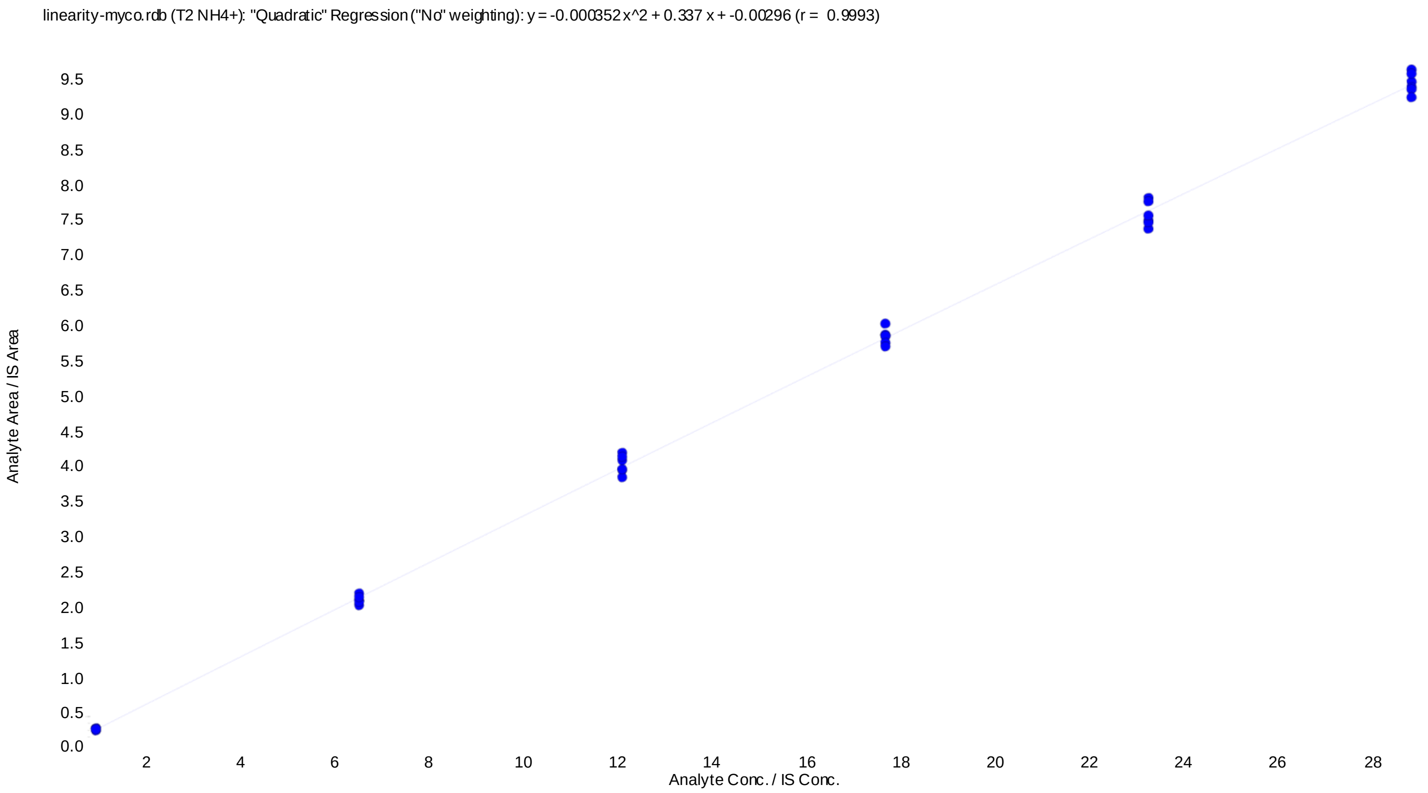

Figure S4. T-2 toxin calibration curve for method linearity.

Analyte Name: HT2 NH4+
Internal Standard: T2-C13MH4+

**Data File**	DataMycotoxin.wiff	**Result Table**	linearity-myco.rdb
**Acquisition Date**	6/8/2017 12:35:18 AM	**Algorithm Used**	IntelliQuan
**Acquisition Method**	mycodraft2.dam	**Instrument Name**	API 4000
**Project**	Default		

Regression Equation: y = -1.77e-005 x^2 + 0.0903 x + 0.00348 (r = 0.9994)

Expected Concentratio n	Number of Values	Mean Calculated Concentratio	% Accuracy	Std. Deviation	%CV
1.55	6	1.55	100.0	0.07	4.5
10.9	6	10.88	99.9	0.36	3.3
20.1	6	19.93	99.1	0.53	2.7
29.4	6	29.74	101.2	0.48	1.6
38.8	6	39.02	100.6	1.17	3.0
48	6	47.63	99.2	1.43	3.0

Printing Time: 01:23:04 AM
Printing Date: Tuesday, July 30, 2019

linearity-myco.rdb (HT2 NH4+): "Quadratic" Regression ("1 / (x * x)" weighting): y = -1.77e-005 x^2 + 0.0903 x + 0.00348 (r = 0.9994)

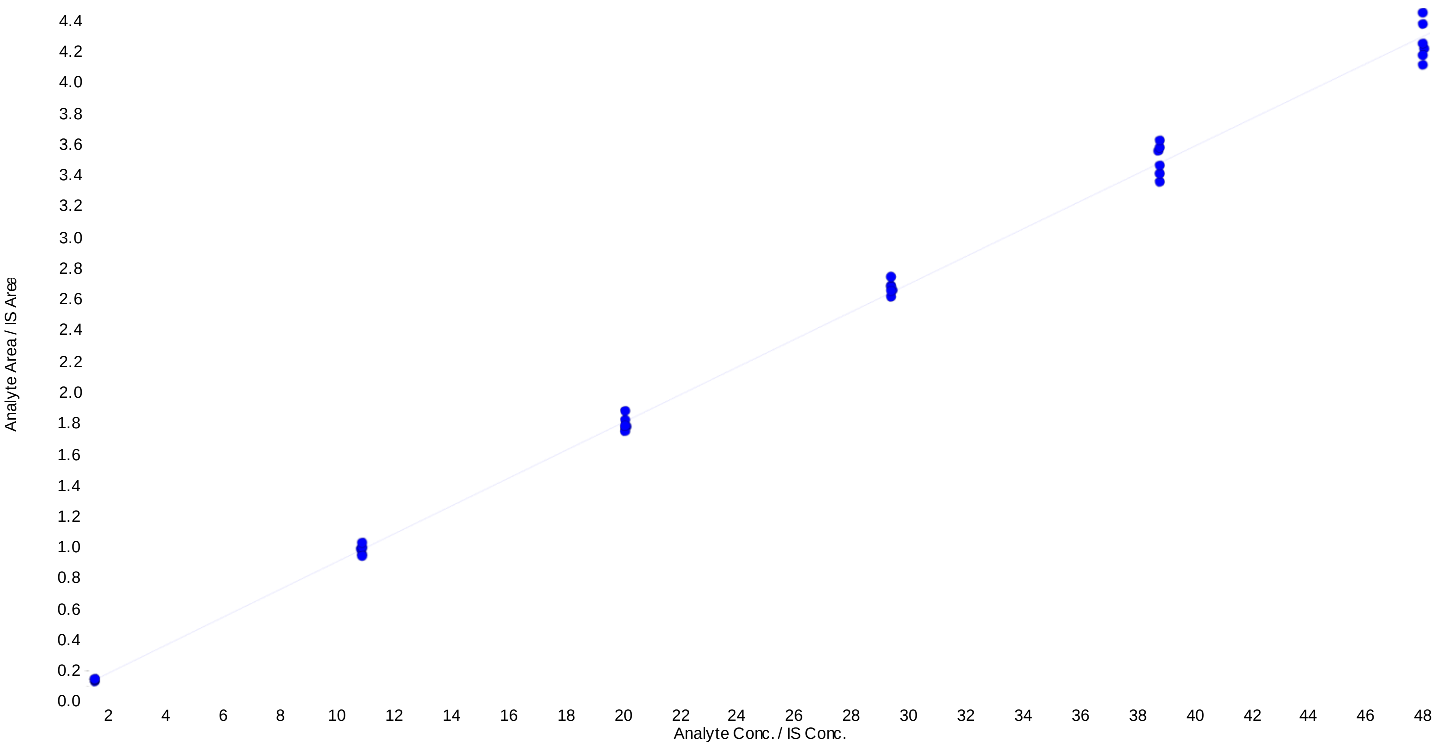

Figure S5. HT-2 toxin calibration curve for method linearity.

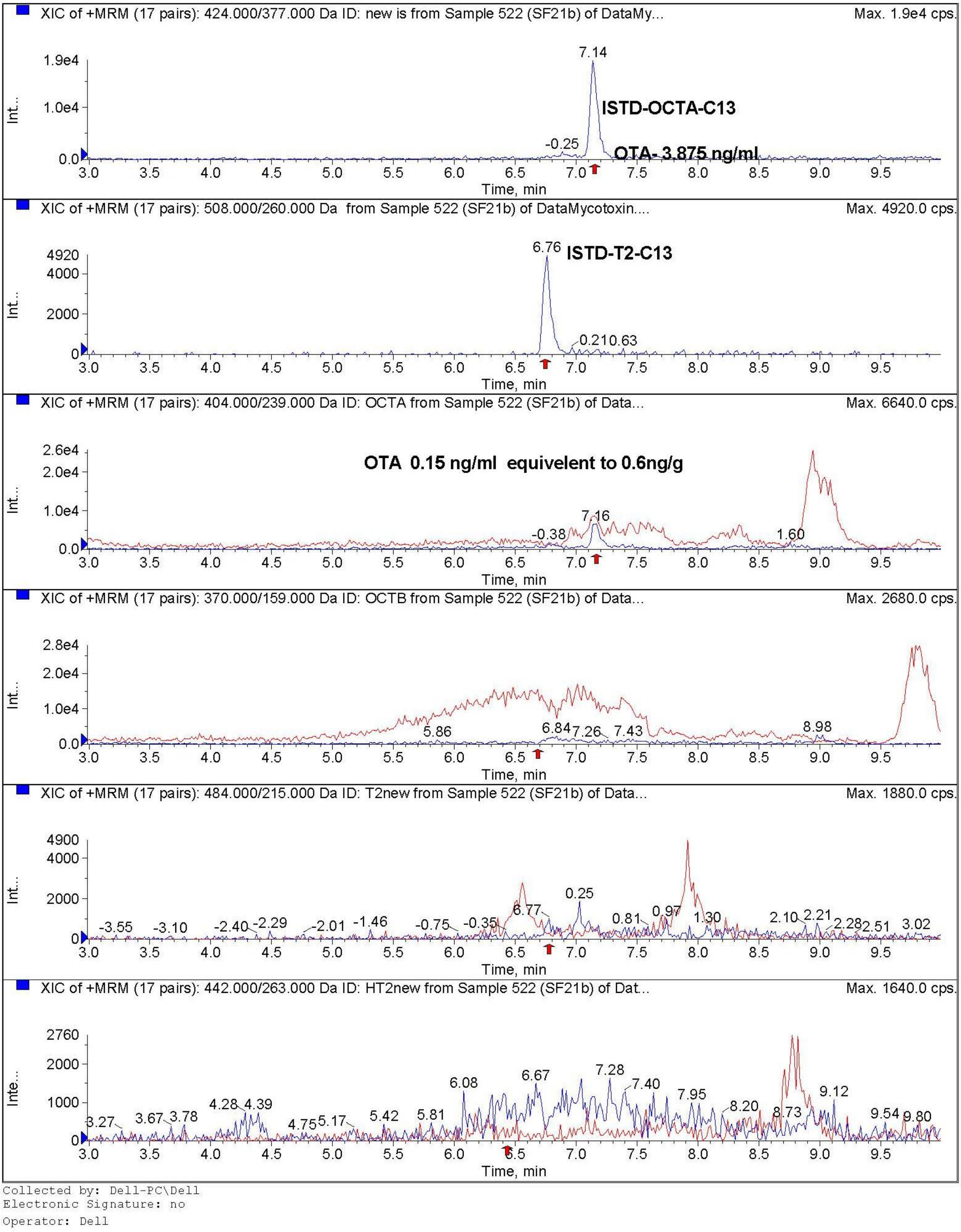

Figure S6. Chromatogram of positive sample (0.6 ng·g^{-1}).

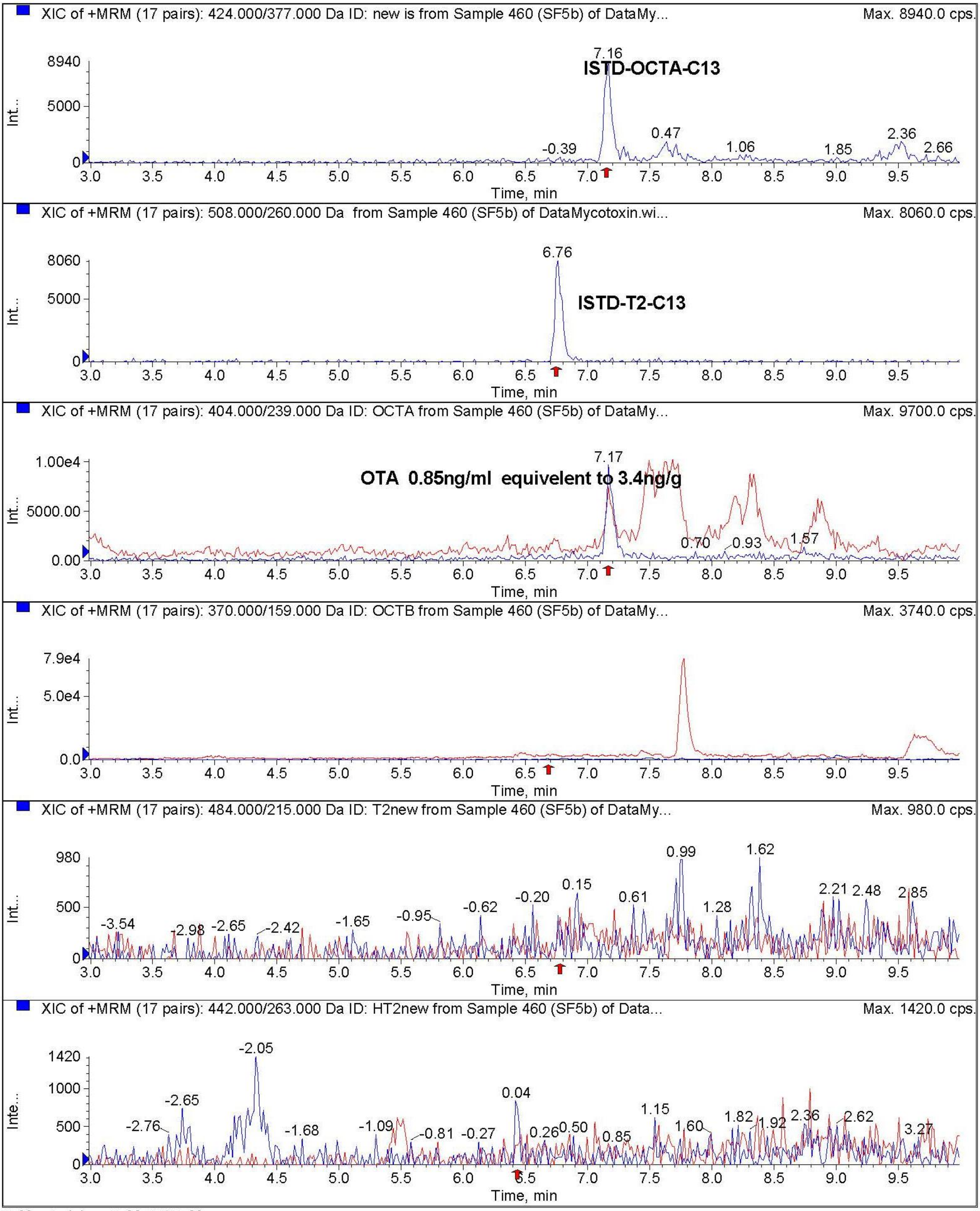

Collected by: Dell-PC\Dell
Electronic Signature: no
Operator: Dell

Figure S7. Chromatogram of positive sample (3.4 ng·g^{-1}).

References

1. Rasmussen, P.H.; Nielsen, K.F.; Ghorbani, F.; Spliid, N.H.; Nielsen, G.C.; Jorgensen, L.N. Occurrence of different trichothecenes and deoxynivalenol-3-β-d-glucoside in naturally and artificially contaminated Danish cereal grains and whole maize plants. *Mycotoxin Res.* **2012**, *28*, 181–190.
2. Klötzel, M.; Lauber, U.; Humpf, H.-U. A new solid phase extraction clean-up method for the determination of 12 type A and B trichothecenes in cereals and cereal-based food by LC-MS/MS. *Mol. Nutr. Food Res.* **2006**, *50*, 261–269.
3. Annunziata, L.; Stramenga, A.; Visciano, P.; Schirone, M.; De Colli, L.; Colograndе, M.N.; Campana, G.; Scortichini, G. Simultaneous determination of aflatoxins, T-2 and HT-2 toxins, and fumonisins in cereal-derived products by QuEChERS extraction coupled with LC-MS/MS. *Anal. Bioanal. Chem.* **2017**, *409*, 5143–5155.
